# Supplementary material for: Resolving the full spectrum of human genome variation using Linked-Reads
Source: Genome Res. 2019 Apr;29(4):635–45. doi: 10.1101/gr.234443.118 (PMC6442396; doi:10.1101/gr.234443.118)
Supplement: Supplemental Material [file supp_gr.234443.118_Supplemental_longranger-2.2.zip › longranger-2.2.tar.bz2!/lr2.2-public/lib/stan/cmdstan/stan/doc/api/html/functions_func_v.html]

Stan: Class Members - Functions


|  |  |
| --- | --- |
|  | Stan  2.5.0  probability, sampling & optimization |


- Main Page
- Related Pages
- Namespaces
- Classes
- Files

- Class List
- Class Index
- Class Hierarchy
- Class Members

- All
- Functions
- Variables
- Typedefs
- Enumerator
- Related Functions

- a
- b
- c
- d
- e
- f
- g
- h
- i
- j
- k
- l
- m
- n
- o
- p
- q
- r
- s
- t
- u
- v
- w
- x
- z
- ~


All Classes Namespaces Files Functions Variables Typedefs Enumerations Enumerator Friends Macros Pages

### - v -

- V()
  : stan::mcmc::base\_hamiltonian< M, P, BaseRNG >
- val()
  : stan::agrad::fvar< T >
  , stan::agrad::var
  , stan::io::cmd\_line
- valid\_value()
  : stan::gm::list\_argument
- validate\_array()
  : stan::gm::validate\_transformed\_params\_visgen
- validate\_dims()
  : stan::io::var\_context
- validate\_identifier()
  : validate\_identifier
- validate\_transformed\_params\_visgen()
  : stan::gm::validate\_transformed\_params\_visgen
- validate\_var\_decl\_visgen()
  : stan::gm::validate\_var\_decl\_visgen
- vals\_i()
  : stan::io::dump
  , stan::io::var\_context
  , stan::json::json\_data
- vals\_r()
  : stan::io::dump
  , stan::io::var\_context
  , stan::json::json\_data
- value()
  : stan::gm::list\_argument
  , stan::gm::singleton\_argument< T >
- values()
  : stan::common::recorder::values< InternalVector >
  , stan::gm::list\_argument
- var()
  : stan::agrad::var
- var\_adaptation()
  : stan::mcmc::var\_adaptation
- var\_context\_factory()
  : stan::common::var\_context\_factory< VARCON >
- var\_decl()
  : stan::gm::var\_decl
- var\_decls\_grammar()
  : stan::gm::var\_decls\_grammar< Iterator >
- var\_resizing\_visgen()
  : stan::gm::var\_resizing\_visgen
- var\_size\_validating\_visgen()
  : stan::gm::var\_size\_validating\_visgen
- vari()
  : stan::agrad::vari
- variable()
  : stan::gm::variable
- variable\_dims()
  : stan::gm::variable\_dims
- variance()
  : stan::mcmc::chains< RNG >
- vector()
  : stan::io::reader< T >
- vector\_constrain()
  : stan::io::reader< T >
- vector\_lb()
  : stan::io::reader< T >
- vector\_lb\_constrain()
  : stan::io::reader< T >
- vector\_lb\_unconstrain()
  : stan::io::writer< T >
- vector\_lub()
  : stan::io::reader< T >
- vector\_lub\_constrain()
  : stan::io::reader< T >
- vector\_lub\_unconstrain()
  : stan::io::writer< T >
- vector\_ub()
  : stan::io::reader< T >
- vector\_ub\_constrain()
  : stan::io::reader< T >
- vector\_ub\_unconstrain()
  : stan::io::writer< T >
- vector\_unconstrain()
  : stan::io::writer< T >
- vector\_var\_decl()
  : stan::gm::vector\_var\_decl
- vectorD()
  : stan::math::LDLT\_factor< stan::agrad::var, R, C >
  , stan::math::LDLT\_factor< T, R, C >
- VectorView()
  : stan::VectorView< T, is\_array, throw\_if\_accessed >
  , stan::VectorView< const double, false, false >
  , stan::VectorView< const T, is\_array, throw\_if\_accessed >
- VectorViewMvt()
  : stan::VectorViewMvt< T, is\_array, throw\_if\_accessed >
  , stan::VectorViewMvt< const T, is\_array, throw\_if\_accessed >
- verbose\_evolve()
  : stan::mcmc::base\_leapfrog< H, P >
- visgen()
  : stan::gm::visgen


---

[ Stan Home Page ]

*© 2011–2014,
Stan Development Team.*
